# Is Gene Flow Promoting the Reversal of Pleistocene Divergence in the Mountain Chickadee (*Poecile gambeli*)?

**DOI:** 10.1371/journal.pone.0049218

**Published:** 2012-11-12

**Authors:** Joseph D. Manthey, John Klicka, Garth M. Spellman

**Affiliations:** 1 Center for the Conservation of Biological Resources, Department of Biology, Black Hills State University, Spearfish, South Dakota, United States of America; 2 Integrative Genomics Program, Black Hills State University, Spearfish, South Dakota, United States of America; 3 Marjorie Barrick Museum of Natural History, University of Nevada Las Vegas, Las Vegas, Nevada, United States of America; Durham University, United Kingdom

## Abstract

The Pleistocene glacial cycles left a genetic legacy on taxa throughout the world; however, the persistence of genetic lineages that diverged during these cycles is dependent upon levels of gene flow and introgression. The consequences of secondary contact among taxa may reveal new insights into the history of the Pleistocene’s genetic legacy. Here, we use phylogeographic methods, using 20 nuclear loci from regional populations, to infer the consequences of secondary contact following divergence in the Mountain Chickadee (*Poecile gambeli*). Analysis of nuclear data identified two geographically-structured genetic groups, largely concordant with results from a previous mitochondrial DNA (mtDNA) study. Additionally, the estimated multilocus divergence times indicate a Pleistocene divergence, and are highly concordant with mtDNA. The previous mtDNA study showed a paucity of sympatry between clades, while nuclear patterns of gene flow show highly varied patterns between populations. The observed pattern of gene flow, from coalescent-based analyses, indicates southern populations in both clades exhibit little gene flow within or between clades, while northern populations are experiencing higher gene flow within and between clades. If this pattern were to persist, it is possible the historical legacy of Pleistocene divergence may be preserved in the southern populations only, and the northern populations would become a genetically diverse hybrid species.

## Introduction

The Pleistocene speciation hypothesis posits that isolation of populations into separate refugia through glacial and interglacial periods was a principal driver of speciation in temperate-adapted taxa [1]. One of the main facets of the Pleistocene speciation hypothesis is the length of time populations must remain in allopatry for lineage divergence (phylogenetic species) or reproductive isolation (biological species) to evolve. The length of time required for either lineage divergence or reproductive isolation to evolve should be different for each pair of isolated populations that experienced unique evolutionary and demographic histories. Demographic shifts (bottlenecks and founder events) associated with tracking habitats into refugia will have a large impact on the rate of stochastic events [2]; whereas dissimilar environmental conditions in separate refugia can impact the strength of selective forces. Therefore, species pairs isolated at approximately the same time during the Pleistocene may be at very different stages of the speciation process or give the appearance of being in different stages as an artifact of the coalescent process. Dozens of phylogeographic studies examining variation in mitochondrial DNA have supported the Pleistocene speciation hypothesis and demonstrated the importance of allopatry in promoting lineage divergence in temperate taxa (see [3] for review). However, relatively few have examined genetic variation at multiple loci (sequence-based nuclear loci), which allows for an evaluation of the microevolutionary processes responsible for driving lineage divergence in allopatry and provides a more complete picture of the speciation process (for some recent examples in birds, see: [4]–[9]).

In western North America, the genetic footprint from the Pleistocene shows consistent broad scale biogeographic patterns, where forest-dwelling taxa exhibit phylogenetic splits between Rocky Mountain (RM) and pacific (Sierra Nevada and Cascade Mountains; PAC) regions, including both intra- and interspecific splits in birds [10]–[15], frogs [16], and mammals [17]. These splits are hypothesized to be the product of isolation in distinct refugia during glacial cycles, followed by expansion into current regions after glacial retreat, often into areas where secondary contact occurs.

Species in western North America that have RM and PAC groups show mixed levels of overlap between phylogenetic groups, ranging from local (e.g. *Poecile gambeli, Dedragapus obscurus*) to somewhat widespread (e.g. *Certhia americana, Catharus ustulatus*). Because all of the aforementioned studies used mitochondrial markers, overlap of clades may indicate hybridization between distinct taxonomic units or sympatry/parapatry of non-interbreeding taxa. In birds (as well as other vertebrates), hybridization following secondary contact is common [18]–[20], with varied consequences. Species may come into secondary contact and form a tension zone, where hybrid abundance and fitness are lower than the parental taxa (e.g. *Pheucticus* [21], *Passerina* [22]). Alternatively, species that come into contact with unrestricted genetic exchange may exhibit secondary intergradation, which may include biased introgression in one direction (e.g. *Vermivora chrysoptera* and *V. pinus* [23]). Finally, secondary contact may lead to speciation through hybridization, resulting in an increase in biodiversity (e.g. *Dendroica auduboni* [24], *Passer italiae* [25]).

Although it is clear that the Pleistocene glacial cycles left a genetic “legacy” on taxa throughout the world [26], these consequences are continually changing through varied levels of gene flow and introgression between lineages. Studying the various consequences of secondary contact among taxa may instill new insights into the history of the Pleistocene’s genetic legacy. Here, we use phylogeographic methods to infer the consequences of secondary contact following divergence in the Mountain Chickadee (*Poecile gambeli*).

**Figure 1:**
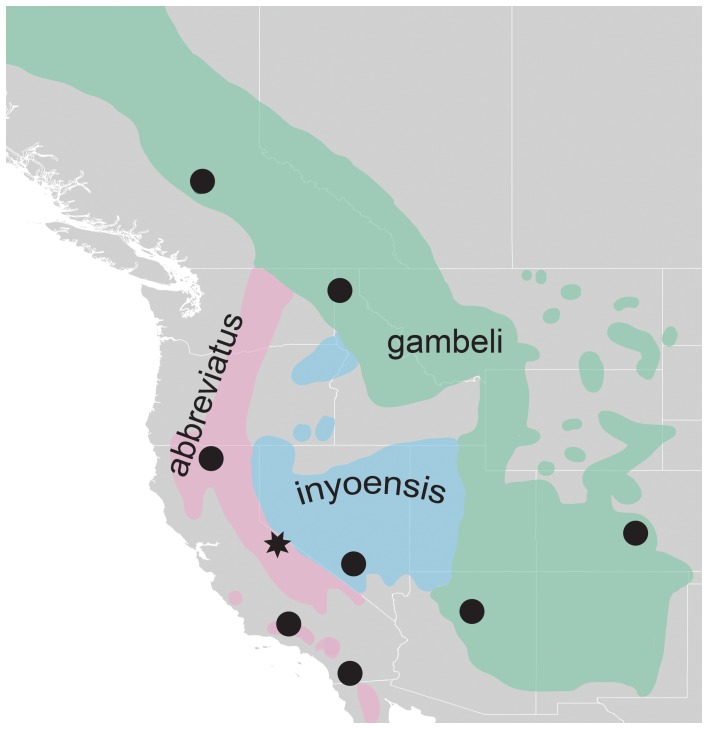
Distribution of *Poecile gambeli*. Breeding distribution of the Mountain Chickadee throughout its range. Different colors represent the three major, widespread morphological groups. The star in eastern California represents the location of a population from [14] that showed both eastern and western mtDNA haplotypes. Dots indicate sampling localities used in this study.

The Mountain Chickadee is a resident of montane coniferous forests in western North America. Up to seven subspecies are recognized, however, they are comprised of three main, widespread morphological groups [27] (Fig. 1): *abbreviatus* (Sierra Nevada and Cascade Mountains populations), *gambeli* (Rocky Mountains) and *inyoensis* (Great Basin). A recent mitochondrial DNA (mtDNA) phylogeographic study [14] identified two genetic lineages: an eastern clade (concordant with the geographic ranges of subspecies *gambeli* and *inyoensis*) and a western clade (concordant with the geographic range of subspecies *abbreviatus*). The two clades are strongly geographically structured, and sampling indicated only a single, local point of contact between clades (Mono County, California [14]; star in Fig. 1). The strong concordance between morphology and genetics led to the proposal that the Mountain Chickadee is comprised of two distinct species, although it was not accepted by the American Ornithologists’ Union [28]. Despite the uncertainty of the taxonomic status of the Mountain Chickadee, it is clear that this taxon is minimally in the process of incipient speciation.

To further elucidate the evolutionary history, reevaluate mtDNA phylogeographic structure, and explore the consequences of secondary contact during incipient speciation, we 1) sequenced 20 nuclear loci from the 3 morphological groups defined by [27], the 2 mtDNA clades identified by [14], and two disjunct populations in southern California, and 2) performed ecological niche modeling of the two mitochondrial lineages. The goals of this study are to: 1) investigate nuclear population structure within and among populations; 2) compare the nuclear genetic patterns and relationships with those of mtDNA; 3) investigate demographic patterns and divergence to infer evolutionary processes driving contemporary patterns; and 4) infer the consequences of secondary contact following divergence.

## Methods

### Ethics Statement

The collection of all animals was conducted under the permission of applicable federal and state agencies. Permits were granted by the following institutions to J.K. and/or G.M.S.: U.S. Department of Fish and Wildlife, Arizona Game and Fish Department, California Department of Fish and Game, Colorado Division of Wildlife, Idaho Fish and Game, Nevada Department of Wildlife and the British Columbia Ministry of Forests, Lands, and Natural Resource Operations. Import and export permits were granted from the United States Department of Agriculture. Birds were collected using shotguns and frozen in the field until museum specimens could be prepared and tissues subsampled and cryogenically stored. Specimen collection was performed following review of the Institutional Animal Care and Use Committee (IACUC) at Black Hills State University (BHSU A-08-001 to GMS). Tissue samples of the specimens from the Museum of Vertebrate Zoology were obtained in accordance with the museum’s loan policy.

### Sampling and Laboratory Procedures

To reduce ascertainment bias in choosing genetic markers [29], we used a single Mountain Chickadee specimen to develop anonymous loci for a majority of the markers used in this study with a protocol identical to [7], described here briefly. Genomic DNA was used for restriction digest and blunt-end cloning followed by sequencing of random inserts. Of 71 successfully sequenced inserts, we removed: redundant sequences, sequences that matched any genes or RNA products in a BLAST (NCBI GenBank Basic Local Alignment Search Tool) search against the Zebra Finch (*Taeniopygia guttata*) genome, or contained repetitive elements. Following these exclusions, 29 sequences were used for developing primers. Of those 29 sequences and developed primers, 14 could be broadly amplified across individuals using polymerase chain reaction (PCR) and were subsequently used in this study (See Table 1 and Supporting Info S1 (Table S1)).

**Table 1:** Summary statistics of genetic markers.

Locus	Chr. #	OAL	#R	Lgth	VS	PIS	H	Indels	D
Pg6	2	255	0	255	11	8	11	0	−1.316
Pg9	Z	274	0	274	16	10	13	0	−1.054
Pg12	15	291	1	186	11	9	13	0	−0.234
Pg13	1	259	0	259	6	5	7	0	−1.329
Pg14	27	270	0	270	6	3	7	0	−1.148
Pg16	18	274	1	260	7	3	6	0	−1.853*
Pg18	20	255	2	138	7	4	7	0	−1.173
Pg47	5	234	1	225	7	3	7	0	−1.827*
Pg48	4	259	1	245	9	4	8	1	−0.561
Pg59	7	280	0	280	12	7	12	0	−1.474
Pg60	2	276	0	276	11	4	11	1	−1.298
Pg61	3	239	1	205	12	8	10	1	−0.790
Pg66	1	280	0	280	7	5	11	0	−0.894
Pg68	17	154	1	150	4	3	5	0	−1.383
CARN	17	432	2	254	10	7	9	0	−1.346
EEF	28	301	5	142	3	2	4	0	−1.335
CLTCL	15	444	1	438	10	4	8	0	−1.141
MUSK	Z	381	0	381	12	8	9	1	0.054
PER	9	413	2	174	11	5	12	0	−1.728
DCOH	6	292	4	95	9	5	9	1	−1.308
		Total	22	4787	181	107	8.95	5	

Original amplicon length (OAL), # recombination events (#R), length after trimming (Lgth), variable sites (VS), parsimony informative sites (PIS), number of haplotypes (H), number of insertion and deletions (indels) and Tajima’s D (D).

Total summary is the average of haplotypes and sum of all other statistics.

**Figure 2:**
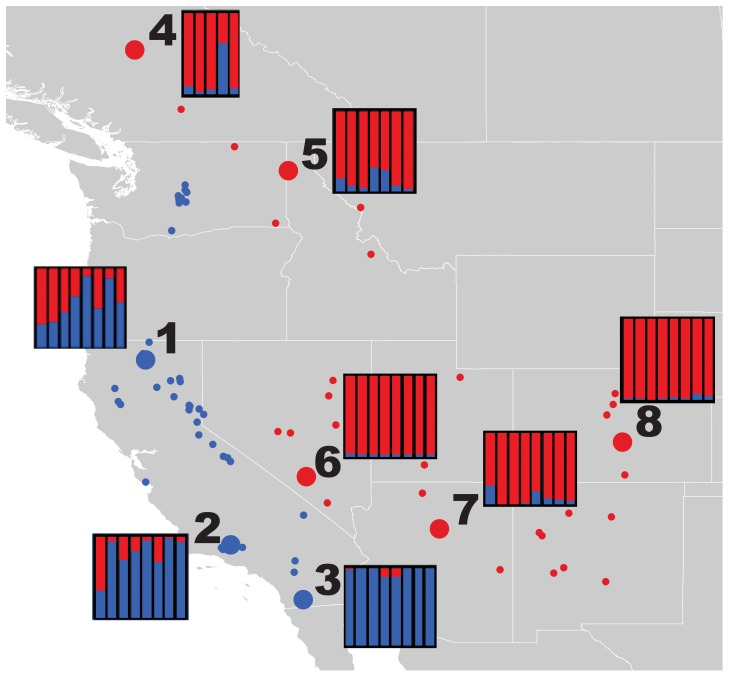
Sampling points and genetic structure in *Poecile gambeli*. Sampling localities, mtDNA assignment of all populations in circles [east or west (red or blue, respectively) from [14]] and STRUCTURE results for all individuals. Each bar represents the assignment probability of an individual to western (blue) or eastern (red) groups. Small white or black circles represent localities (in addition to genetically-sampled populations) used for ecological niche modeling of mitochondrial lineages. Population localities: (1) Siskiyou Co. California, USA (N = 8), (2) Ventura Co. California, USA (N = 8), (3) San Diego Co. California, USA (N = 8), (4) British Columbia, Canada (N = 5), (5) Kootenai Co. Idaho, USA (N = 7), (6) Nye Co. Nevada, USA (N = 8), (7) Coconino Co. Arizona, USA (N = 8), and (8) Routt, Pueblo and Fremont Cos. Colorado, USA (N = 8). Throughout the discussion, populations referred to as northern (or southern) indicate populations 1, 4, 5 (or 2, 3, 6, 7, 8).

Tissue samples of 60 Mountain Chickadee individuals were obtained from eight populations [individuals from [14]; Fig. 2; Supporting Info S1 (Table S2)] representing the three morphological groups defined by [27], the 2 mtDNA clades identified by [14], and two disjunct populations in southern California. Population localities include: Siskiyou Co. California, USA (N = 8; population number 1 in Fig. 2), Ventura Co. California, USA (N = 8; pop. 2), San Diego Co. California, USA (N = 8; pop. 3), British Columbia, Canada (N = 5; pop. 4), Kootenai Co. Idaho, USA (N = 7; pop. 5), Nye Co. Nevada, USA (N = 8; pop. 6), Coconino Co. Arizona, USA (N = 8; pop. 7), and Routt, Pueblo and Fremont Cos. Colorado, USA (N = 8; pop. 8). Hereafter, population labels 1–8 in parentheses refer to population labels in Fig. 2. One sample of the Black-capped Chickadee (*Poecile atricapillus*) was used as an outgroup taxon for genetic structure and phylogenetic analyses.

Total genomic DNA was obtained from specimens used in the mtDNA study [14]. The sequences of 20 loci (introns and anonymous loci) were obtained using PCR amplification with previously characterized primers (introns) and with designed primers (anonymous loci; Supporting Info S1 (Table S1)). PCR amplification of all sequences was carried out in 15 µL reactions and included an initial denaturation period of 10 min at 95°C, with 40 subsequent cycles of 95°C for 30 s, T_A_ for 30 s, and 72°C for 45 s; annealing temperatures varied for each locus from 55–60°C (Supporting Info S1 (Table S1)). This was followed by a 10 min extension at 72°C. PCR products were purified using Exosap-IT (USB Corporation) and sequenced using 10 µL ABI BigDye (Applied Biosystems) sequencing reactions. Sequencing reactions were purified using a standard ethanol-precipitation clean-up followed by sequencing on an ABI 3130 Genetic Analyzer.

Sequences were aligned and edited using Sequencher 4.8 (GeneCodes). Indelligent [30]–[31] was used to help decipher insertions or deletions (indels) in the sequences. Following editing and alignment, sequences were phased in DnaSP [32] with output threshold of 0.7 using algorithms provided by PHASE [33]–[34]. Sequences were checked for recombination using the four-gametes test utilized in DnaSP [32]. If recombination was detected, the most informative, non-recombinant segment was trimmed and used further in analysis.

### Identification of Population Structure

Maximum likelihood (ML) phylogenies were constructed for each locus individually. With the outgroup sequences removed, TOPALi v2.5 [35] was used to select the model of sequence evolution that best fit the sequence data for each locus. Maximum likelihood analyses were performed using a PhyML analysis [36] in TOPALi v2.5 [35] with 100 bootstrap replicates. To quantify exclusive ancestry of populations, we calculated the genealogical sorting index (GSI; www.genealogicalsortingindex.org [37]) for each locus using ML phylogenies followed by 10,000 permutation replicates to test for significance. Because the GSI is a normalized statistic, the GSIs from all loci were used to calculate an ensemble GSI.

To identify the number of populations in equilibrium without a priori input of population assignments, we used the program STRUCTURE [38]–[39]. We used single nucleotide polymorphism (SNP) data from all 20 loci, using an admixture model with correlated allele frequencies between populations. Lambda was inferred by estimating the ln likelihood of k = 1 and allowing lambda to converge. Subsequent runs kept lambda constant using the obtained value from the initial run. Values of k = 1–8 (total number of sampled populations) were tested with ten replicates of each value of k. Each run consisted of a burn-in period of 150,000 steps followed by 1 million iterations. To determine the value of k given our data, we used a combination of the highest log-likelihood of the data and the highest value of ΔK, a statistic calculated using an ad hoc method described by [40]. The ΔK of [39] cannot be calculated at K = 1; however the log-likelihood of the data indicated that K = 1 could be excluded as a possibility.

### Population Analyses

Population analyses were performed for all loci, all sampling localities, as well as all combinations of loci and localities. Genetic diversity per locus was measured by the number of variable sites, number of haplotypes (H), haplotype diversity (H_D_), and nucleotide diversity (π) using ARLEQUIN v3.1 [41]. ARLEQUIN was also used to calculate mismatch distributions [42] by population for each locus to test a model of population growth. The significance of each mismatch distribution was assessed using 1000 replicates of the test. To identify if there is a signature of selection at any loci for each population, Tajima’s D [43] neutrality test was calculated using DnaSP v5 [32].

### Investigation of Population Demographics and Divergence

The Isolation with Migration (IMa) software [44] was used to generate posterior probability distributions for parameters relevant to population demographics and divergence between populations. These comparisons were carried out for each pairwise-comparison of geographically-neighboring populations using the combined mtDNA (ND2 [14]) and nuclear DNA (nuDNA) from all loci. A mutation rate range of 2–3.2% divergence per million years (prior range for ND2∶2.0×10^−8^ to 3.2×10^−8^ mutations/site/year; from: [11], [45]–[46]) was applied to the mitochondrial locus, with the nuDNA allowed to scale to this rate. Although mutation rates may vary for a variety of reasons (e.g. estimation over different timescales [47]), we use a wide prior for mutation rate and the calibrations used are based on multiple studies of both fossil and biogeographic events, specifically for birds. Following trial runs to identify the appropriate scaled model parameter priors, we ran the program for a burn-in period of 500,000 steps followed by 100–150 million iterations (>60 effective sample size for each parameter). Using results from IMa, we calculated the effective migration rate between populations (2 Nm). If Nm <1 (or 2 Nm <2), there is little effective migration and genetic drift will result in substantial local differentiation [48].

### Ecological Niche Modeling of Mitochondrial Lineage Distributions

Geo-referenced specimen data were obtained from [14] to ensure knowledge of mitochondrial lineage. Additional occurrence points in areas without question of mtDNA lineage were obtained from ORNIS, an online database of avian specimen data from North American museums. Occurrence points within 10 km of each other were omitted to prevent sampling identical raster cells multiple times and reduce spatial bias in modeling. Following omission of nearby points, a total of 70 individuals were used (38 Pacific lineage, 32 Rocky Mountain lineage). 19 bioclimatic layers were used from Worldclim [49]. These layers include means, ranges, and extremes of temperature and precipitation. To reduce the number of layers correlated with each other (R^2^<0.8 among all pairwise comparisons of layers), we used a reduced set of 11 variables (see supporting info). A parallel set of climatic layers was downloaded for the Last Glacial Maximum (LGM), approximately 21,000 years before present. The LGM layers are also available on the Worldclim database, and were generated using the Community Climate System Model (CCSM3 [50]).

To create ecological niche models (ENMs), locality data and environmental layers were used in the program Maxent [51]. Maxent uses locality info to determine environmental variables that are associated with the occurrence points, subsequently predicting the probability of potential distributions. Additionally, even with small sample sizes of occurrence data, the models have been shown to perform well [52]. We ran Maxent for 50 replicates, using the bootstrapping method for all iterations, with each mtDNA lineage of Mountain Chickadee input as a separate taxon. We used default settings for all other parameters, except that we used a random seed to randomize testing points for each iteration. These models trained using contemporary conditions were then projected onto conditions from the LGM. Model performance was measured with the area under the receiver operating curve (AUC), using 20% of the samples as test localities and running fifty replicates. Model output was visualized as the 10% training threshold, or approximately 0.2 log model score for each lineage.

To identify levels of niche overlap between lineages, we calculated Schoener’s D statistic [53] using ENMtools [54]–[55]. Schoener’s D is a measure of niche overlap, with higher values (range 0–1) indicating a higher level of overlap between models. Levels of niche movement were also calculated using Schoener’s D by comparing LGM conditions with contemporary conditions for each lineage. This can be interpreted as a lower score equaling more niche movement within a given lineage.

## Results

**Figure 3:**
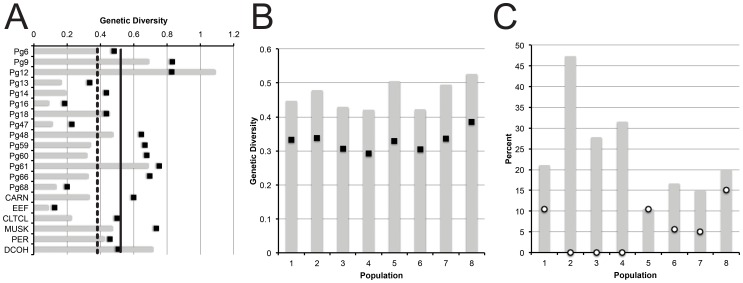
Summary of genetic diversity, mismatch distributions, and neutrality tests. Genetic diversity by locus (A) and by population (B). For both (A) and (B), bars indicate haplotype diversity and dots represent nucleotide diversity. In (B), nucleotide diversity is x100 to match the scale of haplotype diversity. In (C), % of loci per population that are significantly different than the null hypothesis: Bars and dots indicate deviations from mismatch distributions and Tajima’s D neutrality index, respectively.

Twenty loci were sequenced for all 60 Mountain Chickadee samples and one outgroup sample (Black-capped Chickadee). There was an average of 1.10 recombination events detected per locus. Following trimming of recombinant sections, a total of 4,787 base pairs were sequenced for each individual. There were a total of 181 variable sites (107 parsimony informative sites), with an average of 8.95 haplotypes per locus. Average haplotype and nucleotide diversity was 0.517 and 0.00389, respectively. Genetic diversity was not significantly different between introns and anonymous loci (t-test p-values of 0.697 and 0.908 for haplotype and nucleotide diversity, respectively). Per locus variability is reported in Table 1 and Fig. 3A.

### Population Structure and Summary Statistics

**Figure 4:**
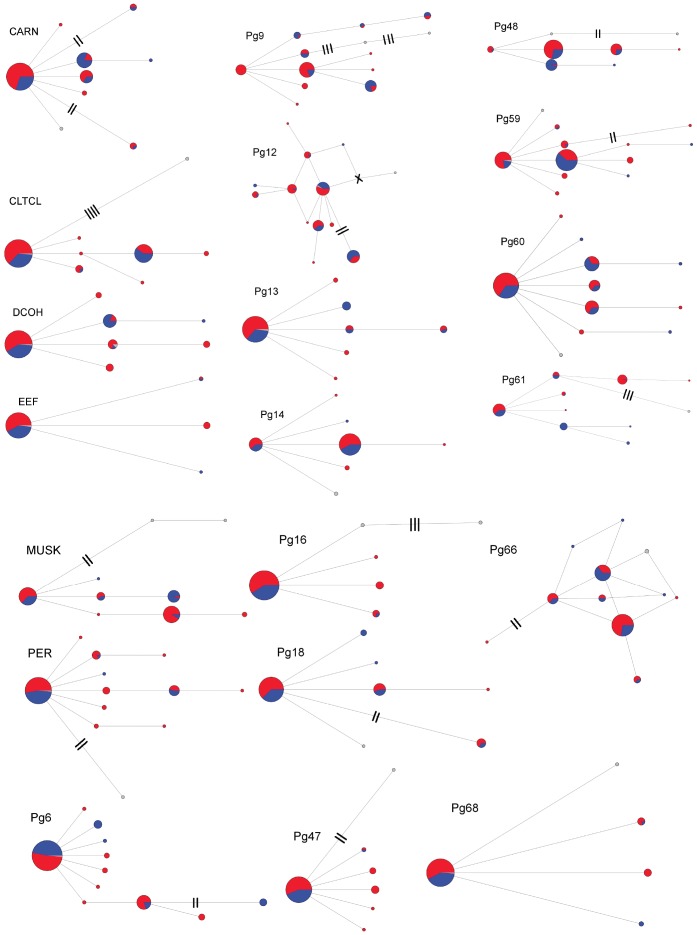
Haplotype networks. Median-joining haplotype networks for all 20 loci used in this study. Haplotypes are sized proportional to frequency (within each marker). Hash marks indicate haplotypes separated by two or more mutations. Blue and red indicate samples with western (pops. 1–3 in Fig. 2) or eastern (pops. 4–8) mtDNA haplotypes, respectively. Gray indicates outgroup samples. Points with an “X” through them (e.g. in Pg12) are inferred haplotypes that are absent in the sampling.

None of the ML gene trees (not shown) exhibited monophyly of any groups [median-joining networks (Fig. 4)]; however, they contained geographically structured patterns of allele frequencies. The ensemble GSI, a direct measure of genetic structure within populations in gene trees, identified low (<0.20), but significant (p<0.04 following Bonferroni correction [56]–[57]) genetic structuring for all but the Colorado population (pop. 8 in Fig. 2; p = 0.175).

The number of populations (K) estimated from the STRUCTURE analysis was k = 2 (Fig. 2; Supporting Info S1 (Fig. S1)), dividing eastern and western populations. The majority of individuals were strongly assigned to one of the groups, with estimated admixture coefficients (Q) greater than 0.8. Thirteen individuals had low (<0.8) Q values, with a large proportion (6/13) of weakly assigned individuals sampled from the Siskiyou population (pop. 1). All population assignments (average of all individuals in the sampled locality), with the exception of the Siskiyou population, were greater than 0.8. Overall, all populations in California sort with a majority assignment to the western clade, and all other populations sort to the eastern clade.

Summaries of genetic diversity, Tajima’s D neutrality index, and mismatch distributions are presented in Figs. 3B, 2C, with all population by locus statistics reported in Supporting Info S1 (Tables S3 and S4). All populations exhibited a similar amount of haplotype (range: 0.418–0.526) and nucleotide (0.00293–0.00385) diversity. Interestingly, the population with the highest genetic diversity (Colorado, pop. 8; Figs. 3B, 2C) was the only population without significant population structure in the GSI analysis. Populations from Ventura, California (2), San Diego, California (3) and British Columbia (4) exhibited the greatest deviationfrom the pattern of an expanding population (Fig. 3C). These were the only populations to not exhibit any deviations from neutrality (Tajima’s D; Fig. 3C). In general, all populations showed little evidence for selection (maximum = three deviations from neutrality in pop. 8). The two disjunct populations in southern California (2 and 3) exhibited the highest GSI values among all populations and were among the top localities in deviating from the signature of an expanding population. Average F_ST_ values (across all loci) range from 0.0033 to 0.0044 between populations and have no correlation with direct geographic distance between populations (R^2^ = 0.0004).

### Patterns of Migration, Population Sizes and Divergence

**Figure 5:**
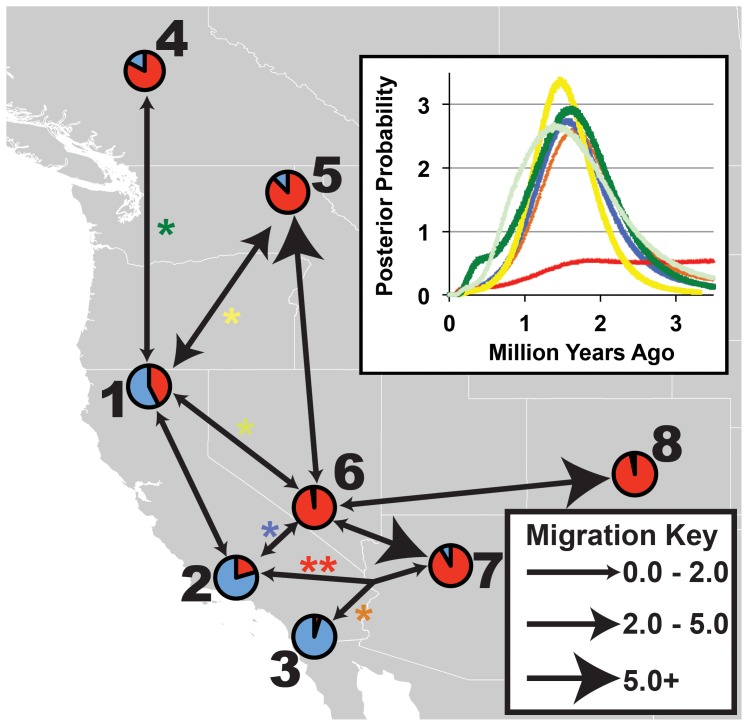
Patterns of migration and divergence between populations in *Poecile gambeli*. Results from coalescent-based isolation with migration (IMa) analyses. Population pie charts shown as a proportion of clade assignment (from Fig. 2). Effective migration (2 Nm) represented by arrows between populations (see migration key). Inset shows divergence time between clades (pairwise comparisons for inset marked with an asterisk on map, asterisks and posterior distributions of inset are colored to indicate population comparisons). The single comparison that reached a plateau in divergence time estimates is marked with a double asterisk. Throughout the discussion, populations referred to as northern (or southern) indicate populations 1, 4, 5 (or 2, 3, 6, 7, 8).

IMa results are summarized in Fig. 5 and Supporting Info S1 (Table S5). Results are reported as highest point estimates and 95% highest probability density (HPD). Divergence between eastern and western populations (population comparisons with an asterisk in Fig. 5) agreed between pairwise comparisons (inset of Fig. 5), with the greatest overlap in divergence curves at ∼1.60 million years ago. A single comparison (between pops. 2 and 7) reached a plateau and failed to converge to zero; however, this comparison reached the plateau at approximately the same peak of the other population comparisons. Between clades, in all comparisons, populations show an increase in effective population size compared to a common ancestor. Within the eastern clade the pattern is the same; however, in the western clade, the comparison between pops. 1 and 2 indicates a population contraction in pop. 2 compared to the ancestral population size, although the HPDs largely overlap.

Effective migration (2 Nm) was, in general, low (2 Nm <2; Fig. 5) between the eastern and western clades. However, migration was high (2 Nm >2) between Siskiyou, California and Idaho (pops. 1 and 5, respectively). In the western clade, migration was low between northern and southern California (Fig. 5). In the eastern clade, migration was low into, but high out of the Great Basin (pop. 6; Fig. 5).

### Ecological Niche Modeling of Mitochondrial Lineages

**Figure 6:**
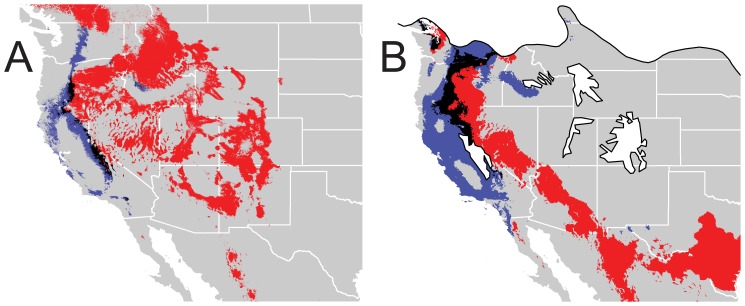
Distributional modeling of *Poecile gambeli* mitochondrial lineages for contemporary and Last Glacial Maximum conditions. Potential niche space models for contemporary (A) and LGM (B) periods. Blue indicates space of western clade, red of eastern clade, and black of areas of overlap between clades. In (B), white space outlined with thick black is indicative of estimated glaciated areas.

ENMs of contemporary and LGM conditions are shown in Fig. 6. Models for the ENMs performed well (mean test AUC = 0.976 and 0.925 for the PAC and RM lineages, respectively); additionally, the combined models together matched the current distribution of the Mountain Chickadee well.

Contemporary ENMs have little overlap between lineages (Schoener’s D = 0.132), while LGM ENMs have relatively higher overlap (D = 0.226). The models with an applied threshold identify two regions of overlap between models: 1) in Mono County, California (population with mixed haplotypes in mtDNA study), and 2) near the Siskiyou County, California population sampled in this study, the population with the weakest nuclear genetic structure. Through glacial cycles, the RM lineage has a high degree of geographic displacement (D = 0.248) while the PAC lineage is more stable (D = 0.565). Size of potential niche space is more stable in the RM lineage (contemporary/LGM space = 1.114) than the PAC lineage (0.257) through time.

## Discussion

### Pleistocene Driven Divergence between Mountain Chickadee Lineages

This study identified two major phylogeographic lineages in the Mountain Chickadee. These lineages correspond with previously identified morphological groups [26] and mtDNA phylogroups [14]. The mtDNA from [14] identified extremely strong lineage sorting, with only a single population (in Mono County, California) possessing haplotypes from both lineages. Similar to the mtDNA, most individuals (all but 13) and populations in the nuclear study (pops. 2, 3, 4, 5, 6, 7, and 8) identified strong phylogeographic structure to either the eastern or western lineages. However, in contrast to the mtDNA study, one population (pop. 1) showed mixed genetic structuring, with many individuals showing ambiguous lineage assignment. The estimated multilocus divergence times indicate a Pleistocene divergence (∼1.6 mya, with the 95% HPD largely overlapping with the Pleistocene; Fig. 5) and are highly concordant with divergence estimates inferred from mtDNA [14]. These findings confirm the importance of the Quaternary glacial cycles in shaping geographic variation in this taxon. Additionally, none of the nuclear loci show fixed polymorphisms between lineages (Haplotype Networks; Fig. 4), suggesting large effective population sizes resulting in a lack of complete lineage sorting. The persistence of large relatively stable populations sizes through glacial cycles within the Mountain Chickadee is supported by several results. First, coalescent-based analyses indicate large population sizes in all populations (generally hundreds of thousands; Supporting Info S1 (Table S5)) are the best fit to the multilocus data. Second, all sampled populations have similarly high levels of genetic diversity (Fig. 3) with few fixed haplotypes (0–10% of loci fixed) within populations. The observed proportion of fixed haplotypes are low compared to other resident, montane avian species with similar genetic sampling schemes (∼20 neutral loci), including *Certhia americana* (5–45% loci fixed within populations; [7]) and *Sitta carolinensis* (31–63%; [9]), suggesting that large population sizes have maintained diversity in the Mountain Chickadee. Third, large areas of contiguous suitable climatic niche space (Fig. 6) could have supported many large and interbreeding populations during glacial and interglacial periods. Fourth, contemporary census data indicates the Mountain Chickadee is one of the most common birds in montane coniferous forests in western North America [58] and if past niche models are remotely accurate the potential remains for large populations in the past.

The strongest nuclear genetic structuring in the western clade is in the Transverse and Peninsular ranges of southern California. This pattern of genetic structure between northern and southern populations in California corroborates mtDNA results, where southern California individuals showed a pattern of nested monophyly within the western clade. Coalescent-based analyses suggest low effective migration between these and other populations, and provide a divergence time of approximately 54 thousand years ago (33.9–364.9 tya 95% HPD) from the northern California population. This time period corresponds with the isolation of southern California mountain ranges by the expansion of the Sonoran and Mohave desert vegetation [59]–[61] and is concordant with many mtDNA genetic splits between avian phylogroups in California [11], [62]. Additionally, this is similar to the nuclear pattern found in *Certhia americana*, in which populations of southern and coastal California showed the strongest genetic isolation in western North America [7]. The signature of isolated populations in southern California is additionally identified in analyses investigating population sizes. Mismatch distributions show the most deviations from the signature of an expanding population in the southern California populations (pops. 2 and 3) and coalescent-based demographic analyses (in IMa) identify a relatively lower increase in effective population size in southern California from an ancestral effective population size (Supporting Info S1 (Table S5)).

### Pleistocene Divergence with Ongoing Gene Flow

Following divergence in the Pleistocene, the two main lineages of Mountain Chickadee have experienced ongoing gene flow [14]. In areas where contact is likely (northern areas between clades) we find a large number of individuals with poor assignment probabilities to either lineage (Fig. 2). Interestingly, the ecological niche models with a threshold applied (as shown in Fig. 6A) identify the areas around Siskiyou County, California (pop. 1) as overlapping between lineages. In the Mountain Chickadee, the strength of nuclear genetic structure appears coincident with the amount of gene flow with other populations. The populations with the strongest genetic structuring (Fig. 2), in southern California and in the Great Basin, have little-to-no incoming gene flow from other populations (2 Nm <2). In contrast, populations in northern California and northern areas of the eastern clade have the weakest population genetic structure, resulting from comparatively (compared with other pairwise population comparisons) high levels of gene flow between populations from different clades (2 NM >2; Fig. 5). Ecological niche models of both past (Fig. 6B) and present (Fig. 6A) seem to corroborate these patterns of gene flow; during both the present and the LGM, southern areas with the strongest genetic structuring appear far from any suitable habitat of the alternate lineage while the opposite is true of northern populations.

The observed pattern of gene flow among populations within and between clades creates a unique pattern of population connectivity. Populations of Mountain Chickadee from the southern extremes of the species range (Peninsular and Transverse mountain ranges of southern California, southern Great Basin, and southern Rocky Mountains) are not exchanging genes with populations from different clades or populations from the same clade. Alternatively, northern populations appear to be exchanging genes with populations from both clades, which may indicate an unsampled hybrid zone between lineages of the Mountain Chickadee. If this pattern were to persist, it is possible the historical legacy of Pleistocene divergence may be preserved in the southern populations only and the northern populations would become a genetically diverse hybrid species [24].

The frequency of gene flow between populations from the two clades was somewhat surprising given the paucity of sympatry observed in the mtDNA data [14]. Sympatry of haplotypes from the most divergent mtDNA clades was observed in a single population from the Mono Crater area of California. The discrepancy between nuclear gene flow and minimal mtDNA sympatry could arise via several processes, including: 1) male-mediated gene flow; 2) nuclear introgression is more common than mtDNA introgression; 3) the amount of sampling did not allow mtDNA to identify additional areas of sympatry (i.e., sparse sampling in central and eastern Oregon and Washington; and 4) an unsampled hybrid zone between lineages which has a larger width for nuclear DNA than mtDNA.

Male-mediated gene flow has been documented in birds; however, in general, males tend to be more philopatric than females [63]–[64]. Interestingly, other bird species that exhibit male-biased gene flow include species that form social groups, like the Mountain Chickadee, such as the White-throated Magpie-jay (*Calocitta Formosa* [65]) and the Siberian Jay (*Perisoreus infaustus* [66]). Male-mediated gene flow could be facilitated by several mechanisms. First, it could be the result of longer natal dispersal in males than females. In Mountain Chickadees, natal dispersal has been observed to neighboring territories, however, in these studies individuals often moved beyond study areas and were not recorded again, suggesting some individuals likely disperse farther [58]. Longer natal dispersal in males could, as was suggested by [66] for the Siberian Jay, be caused by males unable to establish territories nearby natal sites dispersing farther distances than average birds. In this study, however, the two Z-linked loci (MUSK and Pg9) do not appear to have decreased genetic structure between lineages (i.e. signature of male-biased gene flow), with F_ST_ values in the range of the autosomal markers. Because of these results, although only based on two loci, we feel male-mediated gene flow is unlikely between lineages of *Poecile gambeli*.

Alternatively, nuclear gene flow with a lack of mtDNA introgession may be observed because of reduced “hybrid” fitness in the females (i.e. Haldanes’s rule [67]–[68]); this would cause nuclear introgression to be more likely across the lineage boundaries than would mtDNA or sex-linked introgression, a pattern which has been shown in other avian taxa [22], [69]–[70]. Additional examination of the zone of contact between the two Mountain Chickadee lineages will help determine whether the limited sympatry of mtDNA lineages compared to nuDNA is a sampling artifact, the consequence of male mediated dispersal, or the mode of reproductive isolation (i.e., Haldane’s Rule).

Although it is clear that nuclear gene flow between clades is occurring in northern areas; the evolutionary timeframe and mechanism of gene flow is not certain. Our data, combined with mtDNA [14] indicate that the Mountain Chickadee lineages diverged during the Pleistocene glacial cycles and gene flow has occurred across clade boundaries between northern populations. Modeling of lineage distributions (Fig. 6) suggests that the two clades would have remained in contact during glacial and interglacial periods with the possibility of even more extensive contact during glacial maxima. The opportunity for persistent contact over the past two million years suggests the two lineages of Mountain Chickadee may have diverged via parapatric speciation or that the two lineages have experienced transient periods of geographic isolation followed by periods of partial contact. The ability to discern between rates of historical versus contemporary gene flow in the Mountain Chickadee would enhance our understanding of the system, but the timing of migration events is not fully identifiable using current coalescent-based methods [71]. However, if gene flow has been persistent (occurring since divergence) between Mountain Chickadee lineages, we would expect to see more extensive evidence of mixing among populations near contact zones. Thus, we hypothesize either 1) gene flow between lineages is a recent phenomenon, 2) a certain degree of reproductive isolation has evolved between lineages that has limited gene flow between lineages, or 3) a combination of recent gene flow and limited reproductive isolation between lineages. A focused study of the nature of hybridization between lineages in the zone of contact would help discern among these hypotheses.

This study brings further evidence to the consequences of Pleistocene effects on biogeographic patterns of birds. Two major reviews attributed the majority of avian intraspecific phylogeographic breaks to Pleistocene events [72] while most avian interspecific breaks dated to the Pliocene [73]. Klicka and Zink [73] suggested that evidence for subspecies and phylogroup divergence due to the Pleistocene was more likely than traditionally recognized sister species. Additionally, Avise and Walker [72] (p. 462) stated that the Pleistocene’s effects on avian species “will depend primarily on whether environmental conditions over the next two million years are conducive to fostering the survival and continued evolutionary divergence between the intraspecific phylogeographic assemblages so evident in many of today’s avifauna.” This study indicates that the conditions required for continued divergence between Mountain Chickadee lineages during incipient speciation may be eroding, and that contact is promoting gene flow in areas where the two clades are sympatric. These conditions may be exacerbated due to global climate change; shifts in climate regimes may increase sympatry between lineages, thereby increasing the level of hybridization or secondary intergradation, as has been shown in another forest-dwelling vertebrate [Flying Squirrels (*Glaucomys sabrinus* and *G. volans*.) [74]].

## Supporting Information

Supporting Info S1 File including all supplementary tables and figures.
(PDF)

Sequences S1 Zip file of sequences too short for GenBank.
(ZIP)
