# Supplementary material for: Is Gene Flow Promoting the Reversal of Pleistocene Divergence in the Mountain Chickadee (Poecile gambeli)?
Source: PLoS One. 2012 Nov 12;7(11):e49218. doi: 10.1371/journal.pone.0049218 (PMC3495768; doi:10.1371/journal.pone.0049218)
Supplement: Supporting Info S1 — File including all supplementary tables and figures. (PDF) [file pone.0049218.s001.pdf]

Supp. Fig. 1 - Structure k determination. Red and blue lines indicate Log P (X | K) and ΔK respectively.

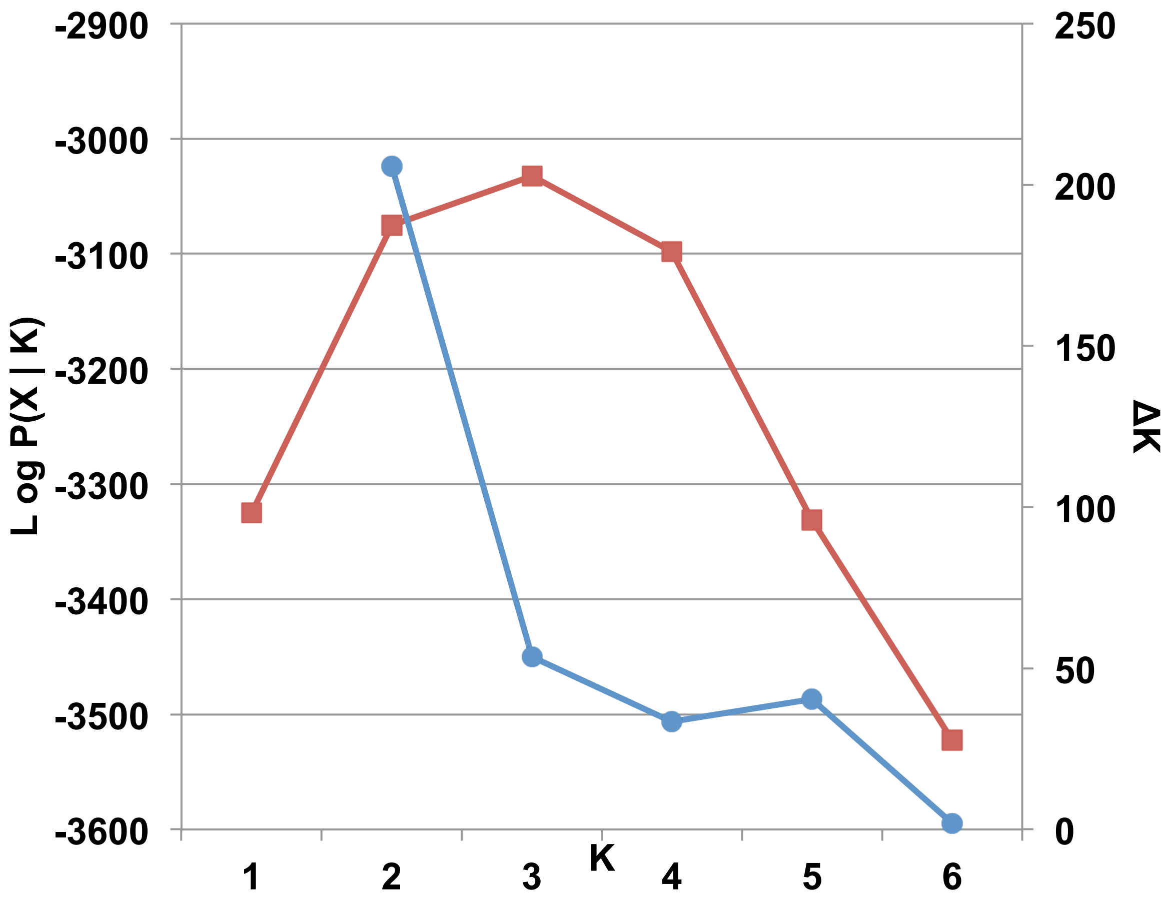

Supplementary Table 1 – List of loci used in study, annealing temperature (T_A), forward and reverse primer sequences for introns and anonymous loci and sequence submission data (GB = Genbank, SF = included as supplementary FASTA file).

Locus	T_A	F Primer	R Primer	Sequence Submission
Anonymous				
Pg6	59	TGTTGCTGTTCTGGTGCAGG	TGAATGGTTTGGCAGTCCCT	GB: JX659167 - JX659286
Pg9	59	CCACCAACCTCAGGCAAAAA	CAGGGCACAGAATGGGACA	GB: JX659287 - JX659391
Pg12	60	GGGCAGCTCAGGTTACTCCC	TGTCTGCAGCTCTCCCGTC	SF
Pg13	60	CCATGGCAACCAGAATTGG	CACAGGTTGCAGGAGGGTTT	GB: JX659392 - JX659513
Pg14	59	TGTCCCTATGGCAGGAGGTG	CTCCATTTCCCTGCCTGCT	GB: JX659514 - JX659635
Pg16	60	CATGCCATTATCCAGCTCCAT	GTGTTGGCCCACTGAGGAA	GB: JX659636 - JX659757
Pg18	58	CCTGTCTCGATGGTCACCAA	CATTCCCCATTTCCAAGCAGT	SF
Pg47	59	CCCTAAAGCCAGCCACAAAC	GCAGAATCAGCGTAGCTGGG	GB: JX659758 - JX659879
Pg48	60	GCTCCAGACCCAGCAAATTG	GACCGTATATGGCATTTAGTGCC	GB: JX659880 - JX660001
Pg59	58	GAGGGTTTTGGGCCAATAATC	GGTAGGCCTGTGGCTGTGAG	GB: JX660002 - JX660123
Pg60	60	CACACTCAGCCTGGCAGGT	AACGGGAGGAGATGTGTTGG	GB: JX660124 - JX660245
Pg61	60	CTGGCCAGTGTTTGCACAGT	TCTTCTGGGTTGAGGCAGGA	GB: JX660246 - JX660367
Pg66	59	TGCATTGCAGGCTGTAGAGAA	CCATGGAGCAGTAACAGGGAG	GB: JX660368 - JX660489
Pg68	60	TCACCAATGCACCAGAGACAG	TTCTCCCATCCTACCCCCTT	SF
Introns				
CARN	55	AGAAAGGCCTGGAGAGGAGAGC	GTCTTCAACCACCAGTCCGAGAG	GB: JX658818 - JX658939
EEF2	55	GAAACAGTTTGCTGAGATGTATGTTGC	GGTTTGCCCTCCTTGTCCTTATC	SF
CLTCL	55	CACCAATGTTCTGCAGAATCCTGA	CCAGCTTATCTTCCTTNAGCCATTTCTC	GB: JX658940 - JX659061
MUSK	55	CTTCCATGCACTACAATGGGAAA	CTCTGAACATTGTGGATCCTCAA	GB: JX659062 - JX659166
PER2	55	CATCTTCAYCCAAATGACAGACC	CCTGATTGGTGAATAGTCAAAAGG	SF
DCOH	55	AGAGCTGTGGGGTGGAACGAGGTGGA	TCRTGGGTGCTCAAGGTGATGTGAAC	SF

Supplementary Table 2 - Marjorie Barrick or Museum of Vertebrate Zoology Museum number (MBM #), Tissue #, Population assignment from Fig. 1, mtDNA haplotype of each individual, and general and specific locality information for each sample used in this study.

MBM #	Tissue #	Pop. #	mtDNA Hap.	Country	State	County	Specific Locality	Lat/Long
MBM12158	dhb5004	**1**	West	USA	CA	Siskiyou	Shasta National Forest, Pilgrim Creek Rd	41°18.9'N/121°59.7'W
MBM12159	dhb5003	**1**	West	USA	CA	Siskiyou	Shasta National Forest, Pilgrim Creek Rd	41°18.9'N/121°59.7'W
MBM12160	dhb5001	**1**	West	USA	CA	Siskiyou	Shasta National Forest, Pilgrim Creek Rd	41°18.9'N/121°59.7'W
MBM12161	dhb5002	**1**	West	USA	CA	Siskiyou	Shasta National Forest, Pilgrim Creek Rd	41°18.9'N/121°59.7'W
MBM12163	dhb4996	**1**	West	USA	CA	Siskiyou	Klamath National Forest, off U.S.97	41°18.9'N/121°59.7'W
MVZ 167850		**1**	West	USA	CA	Siskiyou	N slope Whaleback, 9 mi N 5 mi E Mt. Shasta	41°31.9'N/122°05.4'W
MVZ 167854		**1**	West	USA	CA	Siskiyou	N slope Whaleback, 9 mi N 5 mi E Mt. Shasta	41°31.9'N/122°05.4'W
MVZ 167856		**1**	West	USA	CA	Siskiyou	N slope Whaleback, 9 mi N 5 mi E Mt. Shasta	41°31.9'N/122°05.4'W
MVZ 168174		**2**	West	USA	CA	Ventura	0.5 mi N 1 mi W Frazier Mt.	34°47.2'N/118°59.9'W
MVZ 168175		**2**	West	USA	CA	Ventura	0.5 mi N 1 mi W Frazier Mt.	34°47.2'N/118°59.9'W
MVZ 168176		**2**	West	USA	CA	Ventura	0.5 mi N 1 mi W Frazier Mt.	34°47.2'N/118°59.9'W
MVZ 168178		**2**	West	USA	CA	Ventura	0.5 mi N 1 mi W Frazier Mt.	34°47.2'N/118°59.9'W
MVZ 168179		**2**	West	USA	CA	Ventura	0.5 mi N 1 mi W Frazier Mt.	34°47.2'N/118°59.9'W
MVZ 168180		**2**	West	USA	CA	Ventura	0.5 mi N 1 mi W Frazier Mt.	34°47.2'N/118°59.9'W
MVZ 168182		**2**	West	USA	CA	Ventura	Frazier Mt. 0.5 mi N and 1 mi W	34°47.2'N/118°59.9'W
MVZ 168183		**2**	West	USA	CA	Ventura	1.5 mi N 1.5 mi W Frazier Mt.	34°47.2'N/118°59.9'W
MBM13173	gms605	**3**	West	USA	CA	San Diego	14mi SSE Julian, Mount Laguna, Cleveland NF	32°51.2'N/116°26.1'W
MBM13174	gms606	**3**	West	USA	CA	San Diego	14mi SSE Julian, Mount Laguna, Cleveland NF	32°51.2'N/116°26.1'W
MBM13175	gms612	**3**	West	USA	CA	San Diego	14mi SSE Julian, Mount Laguna, Cleveland NF	32°51.2'N/116°26.1'W
MBM13177	gms610	**3**	West	USA	CA	San Diego	14mi SSE Julian, Mount Laguna, Cleveland NF	32°51.2'N/116°26.1'W
MBM13178	gms611	**3**	West	USA	CA	San Diego	14mi SSE Julian, Mount Laguna, Cleveland NF	32°51.2'N/116°26.1'W
MBM13179	gms608	**3**	West	USA	CA	San Diego	14mi SSE Julian, Mount Laguna, Cleveland NF	32°51.2'N/116°26.1'W
MBM13180	gms609	**3**	West	USA	CA	San Diego	14mi SSE Julian, Mount Laguna, Cleveland NF	32°51.2'N/116°26.1'W
MBM13183	gms607	**3**	West	USA	CA	San Diego	14mi SSE Julian, Mount Laguna, Cleveland NF	32°51.2'N/116°26.1'W
MBM13189	jmd014	**4**	East	CAN	BC		Prince George 26km SSE, Stone Creek	54°07'N/122°30'W
MBM13535	jk03-615	**4**	East	CAN	BC		Williams Lake 20km NNW, Fraser River	52°15'N/122°23'W
MBM13538	jk03-614	**4**	East	CAN	BC		Williams Lake 20km NNW, Fraser River	52°15'N/122°23'W
MBM13539	jk03-612	**4**	East	CAN	BC		Williams Lake 20km NNW, Fraser River	52°15'N/122°23'W
MBM13540	jk03-613	**4**	East	CAN	BC		Williams Lake 20km NNW, Fraser River	52°15'N/122°23'W

MBM13191	jmd018	**5**	East	USA	ID	Kootenai	Cour d'Alene 25mi NNW	47°59'N/116°58'W
MBM13192	jmd019	**5**	East	USA	ID	Kootenai	Cour d'Alene 25mi NNW	47°59'N/116°58'W
MBM13530	jmd022	**5**	East	USA	ID	Kootenai	Cour d'Alene 25mi NNW	47°59'N/116°58'W
MBM13651	gms837	**5**	East	USA	ID	Kootenai	Cour d'Alene 25 mi NNW	47°59'N/116°58'W
MBM13653	gms838	**5**	East	USA	ID	Kootenai	Cour d'Alene 25 mi NNW	47°59'N/116°58'W
MBM13745	gms839	**5**	East	USA	ID	Kootenai	Cour d'Alene 25 mi NNW	47°59'N/116°58'W
MBM13748	gms840	**5**	East	USA	ID	Kootenai	Cour d'Alene 25 mi NNW	47°59'N/116°58'W
MBM8534	GAV1922	**6**	East	USA	NV	Nye	NV Test Site, Area 19, Pahute Msa	37º15'N/116º20'W
MBM9903	gav2223	**6**	East	USA	NV	Nye	NV Test Site Area 19, Pahute Mesa	37.15'N/116.20'W
MBM9904	gav2233	**6**	East	USA	NV	Nye	NV Test Site Area 19, Pahute Mesa	37.15'N/116.20'W
MBM9920	gav2230	**6**	East	USA	NV	Nye	NV Test Site Area 12, Rainier Mesa	37.12'N/116.13'W
MBM9963	gav2236	**6**	East	USA	NV	Nye	NV Test Site Area 19, Pahute Mesa	37.15'N/116.20'W
MBM9964	gav2249	**6**	East	USA	NV	Nye	NV Test Site Area 19, Pahute Mesa	37.15'N/116.20'W
MBM9987	gav2250	**6**	East	USA	NV	Nye	NV Test Site Area 12, Rainier Mesa	37.12'N/116.13'W
MBM9991	gav2244	**6**	East	USA	NV	Nye	NV Test Site Area 12, Rainier Mesa	37.12'N/116.13'W
MBM9577	DHB3952	**7**	East	USA	AZ	Coconino	San Francisco Peaks, Flagstaff 10 mi NNE	35.21.5'N/111.38'W
MBM9578	DHB3953	**7**	East	USA	AZ	Coconino	San Francisco Peaks, Flagstaff 10 mi NNE	35.21.5'N/111.38'W
MBM9579	DHB3948	**7**	East	USA	AZ	Coconino	San Francisco Peaks, Flagstaff 10 mi NNE	35.21.5'N/111.38'W
MBM9580	DCO87	**7**	East	USA	AZ	Coconino	San Francisco Peaks, Flagstaff 10 mi NNE	35.21.5'N/111.38'W
MBM9582	DCO67	**7**	East	USA	AZ	Coconino	San Francisco Peaks, Flagstaff 10 mi NNW	35.19'N/111.45'W
MBM9701	dco115	**7**	East	USA	AZ	Coconino	San Francisco Peaks, Flagstaff 10 mi NNE	35.21.5'N/111.38'W
MBM9702	dco107	**7**	East	USA	AZ	Coconino	San Francisco Peaks, Flagstaff 10 mi NNE	35.21.5'N/111.38'W
MBM9780	gav2192	**7**	East	USA	AZ	Coconino	San Francisco Peaks, Flagstaff 10 mi NNE	35.21.5'N/111.38'W
MBM12168	dhb5047	**8**	East	USA	CO	Routt	Routt National Forest, Forest Rd.241	40°05.1'N/106°36.5'W
MBM12169	dhb5046	**8**	East	USA	CO	Routt	Routt National Forest, Forest Rd.241	40°05.1'N/106°36.5'W
MBM12269	dhb5115	**8**	East	USA	CO	Pueblo	San Isabel National Forest, Forest Rd.386	38°07.4'N/105°07.5'W
MBM12270	dhb5114	**8**	East	USA	CO	Pueblo	San Isabel National Forest, Forest Rd.386	38°07.4'N/105°07.5'W
	dhb2255	**8**	East	USA	CO	Fremont	Canon City, 12mi NW	38º25'N 105º11'W
	dhb2299	**8**	East	USA	CO	Fremont	Canon City, 12mi NW	38º25'N 105º11'W
	dhb2300	**8**	East	USA	CO	Fremont	Canon City, 12mi NW	38º25'N 105º11'W
	dhb3623	**8**	East	USA	CO	Fremont	Canon City, 12mi NW	38º25'N 105º11'W
	GMS2780	Out	Outgroup	USA	SD	Lawrence	*Poecile atricapilus*	

Supp Table 3 – Number of haplotypes and nucleotide diversity per population by locus.

	Siskiyou (1)		Ventura (2)		San Diego (3)		British Columbia (4)		Idaho (5)		Nevada (6)		Arizona (7)		Colorado (8)	
Locus	H	π	H	π	H	π	H	π	H	π	H	π	H	π	H	π
Pg6	3	0.001471	3	0.002745	2	0.001275	5	0.007320	5	0.005128	2	0.005098	5	0.005523	2	0.001830
Pg9	3	0.002807	5	0.007355	4	0.009465	4	0.003650	5	0.004024	5	0.004775	5	0.004118	5	0.006537
Pg12	7	0.013978	4	0.007572	3	0.006317	6	0.010872	4	0.009217	5	0.007930	6	0.009812	7	0.010125
Pg13	3	0.002284	3	0.002510	3	0.001737	1	-	1	-	3	0.000965	3	0.001384	3	0.003411
Pg14	4	0.002778	2	0.000864	2	0.001204	2	0.000741	2	0.001628	4	0.002778	3	0.002315	3	0.001667
Pg16	2	0.000481	2	0.000897	1	-	1	-	4	0.001648	1	-	2	0.000897	2	0.000897
Pg18	4	0.005193	2	0.002899	4	0.006401	2	0.005153	2	0.001911	3	0.003502	3	0.004167	4	0.006643
Pg47	1	-	2	0.000556	2	0.000556	2	0.000889	3	0.001270	1	-	3	0.002000	3	0.002333
Pg48	3	0.004796	5	0.005340	3	0.005986	3	0.002268	4	0.002736	3	0.002143	3	0.001837	4	0.003605
Pg59	4	0.002500	3	0.002083	3	0.001280	5	0.005079	5	0.003728	5	0.002946	5	0.002768	5	0.004345
Pg60	6	0.005103	4	0.003455	4	0.002758	3	0.001455	6	0.003037	3	0.002515	3	0.003121	5	0.003212
Pg61	3	0.003821	5	0.008618	3	0.003821	2	0.001951	4	0.007880	3	0.004553	4	0.006423	2	0.004878
Pg66	5	0.002857	5	0.003512	6	0.003512	3	0.001825	4	0.002433	5	0.002440	4	0.002619	6	0.004435
Pg68	2	0.001556	2	0.000833	1	-	2	0.001333	2	0.000952	2	0.000833	2	0.000833	3	0.003722
CARN	4	0.002395	5	0.004035	4	0.004626	2	0.000787	3	0.002163	3	0.001903	5	0.003543	7	0.004364
CLTCL	4	0.001560	2	0.002283	2	0.002283	2	0.002435	3	0.002860	3	0.001313	2	0.000571	5	0.001674
DCOH	4	0.005088	2	0.005263	2	0.005263	2	0.002105	4	0.011567	4	0.020351	4	0.006053	3	0.005877
EEF	2	0.000880	1	-	2	0.000880	2	0.002817	2	0.003715	2	0.001761	2	0.001761	2	0.000880
MUSK	4	0.002778	3	0.004856	2	0.002625	3	0.002975	5	0.004615	2	0.002625	4	0.003368	3	0.002428
PER	4	0.005172	2	0.002682	2	0.001437	5	0.009068	5	0.004547	3	0.003209	5	0.004215	5	0.003496
Average	3.6	0.003375	3.1	0.003418	2.8	0.003071	2.9	0.003136	3.7	0.003753	3.1	0.003582	3.7	0.003366	4.0	0.003818

Supp Table 4 – Mismatch distributions and Tajima's D by population for each locus. % Deviant(final row) = percent of loci significantly different from the null hypothesis.

	Siskiyou (1)		Ventura (2)		San Diego (3)		British Columbia (4)		Idaho (5)		Nevada (6)		Arizona (7)		Colorado (8)	
Locus	MM	D	MM	D	MM	D	MM	D	MM	D	MM	D	MM	D	MM	D
Pg6	NS	-1.697*	p = 0.00	-0.654	NS	0.156	NS	0.225	NS	0.131	p = 0.00	0.245	NS	-0.757	p = 0.00	-0.578
Pg9	NS	-0.646	NS	0.800	NS	1.345	NS	-0.359	NS	-0.491	NS	0.267	NS	-0.424	NS	1.397
Pg12	NS	0.283	NS	0.527	NS	0.869	NS	-0.193	NS	0.313	NS	-0.070	NS	0.032	NS	-0.378
Pg13	NS	-1.002	p = 0.05	0.201	NS	-0.649	-	-	-	-	NS	-1.498*	NS	-1.038	p = 0.00	-0.067
Pg14	NS	-0.494	NS	-0.448	NS	0.156	p = 0.05	-1.112	NS	0.842	NS	-0.494	NS	0.095	NS	-0.649
Pg16	NS	-1.162	NS	-0.448	-	-	-	-	NS	-1.671*	-	-	p = 0.05	-0.448	NS	-0.448
Pg18	p = 0.00	-1.269	NS	0.650	NS	-0.836	p = 0.00	0.019	NS	-0.341	NS	-1.349	NS	-1.055	NS	-0.750
Pg47	-	-	p = 0.05	-1.162	p = 0.05	-1.162	p = 0.05	-1.112	NS	-1.481	-	-	NS	-0.649	NS	-0.330
Pg48	p = 0.00	0.869	NS	0.267	NS	1.804	NS	-0.691	NS	-0.886	NS	-0.330	NS	-0.649	NS	-0.067
Pg59	NS	-1.312	NS	-0.082	NS	-1.038	NS	-0.783	NS	-0.560	NS	-0.987	NS	-1.117	NS	0.029
Pg60	NS	-0.994	NS	0.147	p = 0.05	-0.467	NS	-1.401	NS	-1.623*	p = 0.00	0.378	NS	1.085	NS	-0.836
Pg61	NS	0.236	p = 0.00	0.613	NS	0.236	NS	-1.112	NS	1.004	NS	1.404	NS	0.289	NS	1.687
Pg66	NS	0.838	NS	0.254	NS	0.254	NS	1.142	p = 0.05	0.226	NS	-0.708	p = 0.05	0.555	NS	-1.072
Pg68	NS	-0.448	p = 0.05	-1.162	-	-	NS	-1.112	NS	-1.155	NS	-1.162	NS	-1.162	NS	-0.186
CARN	NS	-1.550*	NS	-1.054	NS	-0.079	p = 0.05	-1.112	NS	-1.278	NS	-1.349	NS	-1.327	NS	-1.948**
CLTCL	NS	-0.708	p = 0.05	1.687	p = NS	1.687	NS	1.641	NS	1.004	NS	-1.055	p = 0.05	-1.498	p = 0.00	-1.692*
DCOH	NS	-1.349	p = 0.05	1.309	NS	1.309	p = 0.05	-1.112	NS	-0.886	NS	0.174	NS	-1.055	NS	-0.189
EEF	p = 0.00	-1.162	-	-	NS	-1.162	p = 0.05	-1.112	p = 0.05	-0.341	NS	-1.162	NS	-1.162	NS	-1.162
MUSK	NS	0.495	p = 0.05	1.673	p = 0.05	1.687	NS	0.247	NS	0.410	p = 0.05	1.687	NS	1.216	p = 0.00	0.067
PER	p = 0.00	-0.793	p = 0.00	-0.578	p = 0.00	-1.498	NS	0.445	NS	-1.222	NS	-0.189	NS	-1.692*	NS	-1.550*
%	21.1	10.5	47.4	0.0	27.8	0.0	31.6	0.0	10.5	10.5	16.7	5.6	15.0	5.0	20.0	15.0

Supp. Table 5 – IMa output and 95% HPD. q and t are in thousands. * indicates a run that did not converge for t and had a value that created a plateau.

	q1	q2	qA	t	2Nm1	2Nm2
7 - 6	364.57	51.95	0.16	468.71	10.80	1.60
	202.54 - 875.45	29.39 - 237.80	4.61 - 268.30	131.13 - 1158.65	0.35 - 60.24	0.16 - 33.72
7 - 3	997.91	163.04	8.94	1657.40	0.72	0.70
	623.26 - 1744.91	73.48 - 389.47	12.09 - 891.95	769.61 - 4983.72	0.07 - 6.46	0.13 - 7.10
7 - 2	908.17	238.96	35.01	1571.80	0.78	1.33
	567.22 - 1527.14	126.33 - 518.01	12.68 - 826.48	677.45 - 4233.41	0.06 - 7.96	0.30 - 8.77
8 - 6	576.25	125.00	121.25	815.15	18.81	1.66
	349.18 - 4003.79	69.73 - 367.28	10.71 - 707.36	100.80 - 1545.37	3.18 - 332.74	0.11 - 17.87
6 - 5	56.60	278.73	71.37	78.48	0.80	5.76
	29.55 - 179.81	151.35 - 1008.00	6.25 - 137.82	39.74 - 507.92	0.07 - 20.30	0.30 - 95.69
6 - 2	357.93	274.10	4.31	6809.94*	0.57	1.22
	198.49 - 654.64	155.75 - 543.68	33.08 - 2751.49	746.72 - 6748.00	0.08 - 5.83	0.17 - 6.28
1 - 4	689.03	887.64	6.59	1620.29	1.55	0.82
	367.57 - 1421.54	479.23 - 2287.45	14.49 - 936.72	440.37 - 3238.48	0.14 - 15.88	0.08 - 21.43
1 - 5	588.67	875.63	1.61	1464.29	2.16	2.64
	301.71 - 1243.05	470.67 - 1741.88	8.05 - 659.21	728.00 - 2562.10	0.22 - 18.59	0.22 - 23.30
1 - 6	779.65	256.97	2.01	1390.36	0.65	1.06
	342.19 - 1667.99	126.81 - 717.25	7.38 - 931.95	582.91 - 3876.23	0.04 - 11.57	0.16 - 11.13
1 - 2	419.21	112.80	190.92	54.16	0.01	0.00
	297.99 - 9651.93	64.31 - 357.25	56.23 - 306.75	33.92 - 364.88	0.24 - 575.07	0.05 - 16.34

Supp. Info. – Bioclimatic layers used in ecological niche modeling and descriptions of each variable.

Variable	Description
Bio1	Annual mean temperature
Bio2	Monthly temperature range
Bio4	Temperature seasonality
Bio5	Max temperature warmest month
Bio6	Min temperature coldest month
Bio9	Mean temperature driest quarter
Bio12	Annual precipitation
Bio15	Precipitation seasonality
Bio17	Precipitation driest quarter
Bio18	Precipitation warmest quarter
Bio19	Precipitation coldest quarter
